# Cell-Free Protein Expression under Macromolecular Crowding Conditions

**DOI:** 10.1371/journal.pone.0028707

**Published:** 2011-12-08

**Authors:** Xumeng Ge, Dan Luo, Jianfeng Xu

**Affiliations:** 1 Arkansas Biosciences Institute and College of Agriculture and Technology, Arkansas State University, Jonesboro, Arkansas, United States of America; 2 Department of Biological and Environmental Engineering, Cornell University, Ithaca, New York, United States of America; Shantou University Medical College, China

## Abstract

**Background:**

Cell-free protein expression (CFPE) comprised of *in vitro* transcription and translation is currently manipulated in relatively dilute solutions, in which the macromolecular crowding effects present in living cells are largely ignored. This may not only affect the efficiency of protein synthesis *in vitro*, but also limit our understanding of the functions and interactions of biomolecules involved in this fundamental biological process.

**Methodology/Principal Findings:**

Using cell-free synthesis of *Renilla* luciferase in wheat germ extract as a model system, we investigated the CFPE under macromolecular crowding environments emulated with three different crowding agents: PEG-8000, Ficoll-70 and Ficoll-400, which vary in chemical properties and molecular size. We found that transcription was substantially enhanced in the macromolecular crowding solutions; up to 4-fold increase in the mRNA production was detected in the presence of 20% (w/v) of Ficoll-70. In contrast, translation was generally inhibited by the addition of each of the three crowding agents. This might be due to PEG-induced protein precipitation and non-specific binding of translation factors to Ficoll molecules. We further explored a two-stage CFPE in which transcription and translation was carried out under high then low macromolecular crowding conditions, respectively. It produced 2.2-fold higher protein yield than the coupled CFPE control. The macromolecular crowding effects on CFPE were subsequently confirmed by cell-free synthesis of an approximately two-fold larger protein, *Firefly* luciferase, under macromolecular crowding environments.

**Conclusions/Significance:**

Three macromolecular crowding agents used in this research had opposite effects on transcription and translation. The results of this study should aid researchers in their choice of macromolecular crowding agents and shows that two-stage CFPE is more efficient than coupled CFPE.

## Introduction

Cell-free (*in vitro*) protein expression (CFPE) has become an invaluable platform for rapid and parallel synthesis of functional proteins [1], [2]. The open nature and high versatility of the CFPE platform enable the production of proteins that are otherwise hard or impossible to express with a cell-based system such as membrane proteins, cell-toxic proteins, isotope-labeling proteins and protein with unnatural amino acids incorporated [3]–[9]. In the post-genomic era, CFPE has become one of the most important high-throughput tools for functional genomics and proteomics [10]–[13].

CFPE reproduces *in vitro* two fundamental biological processes, transcription and translation. It provides an essential platform for the study of genetic information transfer from DNA to protein by overcoming the barrier to *in situ* biomolecular characterization caused by the labile nature of cell wall, membrane and organelles [14]. However, CFPE is routinely carried out in relatively dilute solutions, where a common intracellular feature, macromolecular crowding is neglected. Due to presence of high concentrations (300–400 g/L) of biomolecules such as proteins, nucleic acids, ribosomes and carbohydrates that occupy 20–30% (v/v) of cytoplasmic volume, the intracellular environment of living cells is highly crowded [15], [16]. This can result in surprisingly large qualitative and quantitative effects on both the thermodynamic and kinetic of interactions among biomolecules [15], [17]–[21]. Thus, investigation of CFPE under cell-like macromolecular crowding conditions becomes very important, as it will allow us to better reproduce the fundamental biological process in an *in vitro* setting. For example, it can help design and construct more cell-like synthetic minimal “cells”, in which transcription/translation are most often used as the fundamental basis or served as a “central node” to network other biological processes [22]–[25].

Since macromolecular crowding is a ubiquitous and fundamental feature of all living organisms, there have recently been a surge of interests in studying the effects of macromolecular crowding on various biological processes [16], [26], and in revealing how biomolecules behave under these cell-like excluded volume conditions [21], [27]–[37]. However, only a few studies have been published relevant to the macromolecular crowding effects on CFPE. For example, Sanders et al. studied transcriptional activation of bacteriophage T4 late genes by using such crowding agents as polyethylene glycol (PEG), polyvinyl alcohol, dextran and Ficoll [38]. Nakano et al reported enhanced protein expression by using condensed wheat-germ extract or adding PEG in *E. coli* S30 extract [39], [40]. In contrast, Bakke et al. found that low macromolecular crowding environments were favored by CFPE process and subsequent protein detection [14]. In addition, the association of ribosomal particles with mRNA in the *in vitro* translation was found to increase by the addition of crowding agents [41], but no improvement of translation was demonstrated. CFPE were also carried out under some unusual conditions in which DNA templates were incorporated into DNA hydrogel [42], or entrapped in calcium alginate micro-beads [43] and silica sol-gel [44]. Enhanced transcription and translation were found in all these cases. However those solid matrix environments, though “crowded” as well, are radically different from the liquid-phase macromolecular crowding environment. Up to date, no systematic study on the macromolecular crowding effects on the CFPE has been reported.

The present study provides an extensive investigation of the CFPE under macromolecular crowding conditions by using as a model system the synthesis of a reporter protein *Renilla* luciferase (Rluc, 36 kDa) in the wheat germ (WG) extract-based CFPE system. This is followed by investigation into synthesis of an approximately two-fold larger protein, *Firefly* luciferase (Fluc, 62 kDa), to test the general applicability of macromolecular crowding effects to CFPE. The crowding environments *in vitro* are emulated by three inert macromolecular crowding agents, polyethylene glycol (PEG)-8000, Ficoll-70 and Ficoll-400, which vary in chemical properties, molecular size and morphology. While the PEG-8000 occurs as a flexible long-chain polyethylene glycol with sparse and short branches, the two Ficoll molecules are highly branched copolymers of sucrose that have more spherical and compact structures [45].

## Results

### In vitro transcription under macromolecular crowding conditions

**Figure 1:**
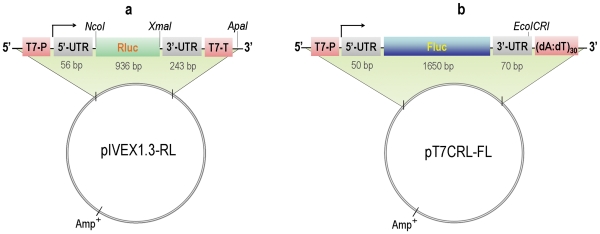
Schematics of the expression vectors used for *in vitro* transcription and translation. (**a**) pIVEX1.3-RL vector harboring Rluc gene; (**b**) pT7CRL-FL vector harboring Fluc gene. For both vectors, gene expression was driven by a T7 promoter. T7-P: T7 promoter; T7-T: T7 terminator; 5′-UTR: 5′-untranslational region; 3′-UTR: 3′-untranslational region.

**Figure 2:**
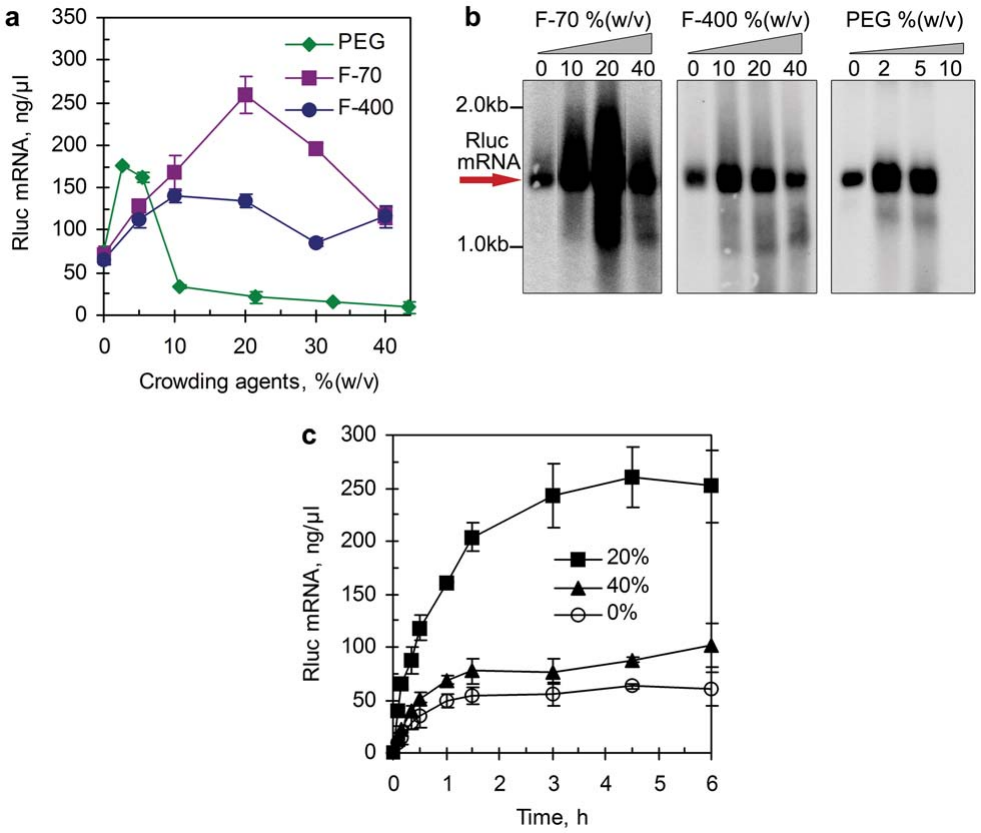
*In vitro* transcription under macromolecular crowding conditions. (**a**) Effect of macromolecular crowding agents, Ficoll-70 (F-70), Ficoll-400 (F-400) and PEG-8000 (PEG) on the Rluc mRNA synthesis from the pIVEX1.3-RL template. Transcriptions were incubated for 3 hr; (**b**) Northern blotting analysis of the Rluc mRNA synthesized by *in vitro* transcription; (**c**) Time course of *in vitro* transcription under macromolecular crowding conditions emulated by Ficoll-70. The initial velocity of transcription (409, 146 and 94 ng/µl/min for reactions with 20%, 40% and 0% (w/v) Ficoll-70, respectively) was calculated based on the synthesis of mRNA during the period of 0–10 min. In panel **a** and **c**, each data point is the mean of triplicate values; error bars indicate the standard deviation from the mean.

We uncoupled the two consecutive processes of CFPE and first investigated the *in vitro* transcription under macromolecular crowding conditions using a pIVEX1.3-RL plasmid harboring Rluc gene (Figure 1). Transcription was enhanced by the addition of each of the crowding agents studied (Figure 2a). The Ficoll-70 showed the most significant effect, increasing the mRNA yield to 260 ng/µl in the presence of 20% (w/v) of Ficoll-70. This represents a 4-fold increase in the mRNA yield compared to transcription in the control dilute solution. The influence of PEG on transcription differed from that of the Ficoll-70 and Ficoll-400, since it dramatically enhanced transcription at low concentrations (0–5.4%, w/v), but quickly became inhibitory when the concentrations of the PEG exceeded 10% (w/v).

Northern blotting analysis detected the integrity of the target mRNA generated from transcription. Figure 2b showed that distinctive Rluc mRNA bands (∼1.4 kb) were detected under different macromolecular crowding condition and the intensity of each band was in good conformity with the mRNA yields measured with the Quant-iT™ RiboGreen® RNA reagent. However, we did find a small amount of truncated mRNA (∼1.0–1.2 kb) generated under higher concentrations of Ficoll-400 (20% and 40%, w/v) or in the presence of PEG.

We then determined the macromolecular crowding effects on the time course (kinetics) of transcription. In the presence of 20% (w/v) of Ficoll-70 transcription proceeded much faster than in the dilute solution with a ∼3-fold increase in the initial transcription velocity (Figure 2c). Furthermore, the crowding agent extended the reaction time from 1.5 hr (as found in the dilute solution) to 4.5 hr. However, when an extremely high concentration of the Ficoll-70 (40%, w/v) was used, transcription slowed down instead compared to that with 20% (w/v) of Ficoll-70, and its kinetics profile was close to that in the dilute solution.

**Figure 3:**
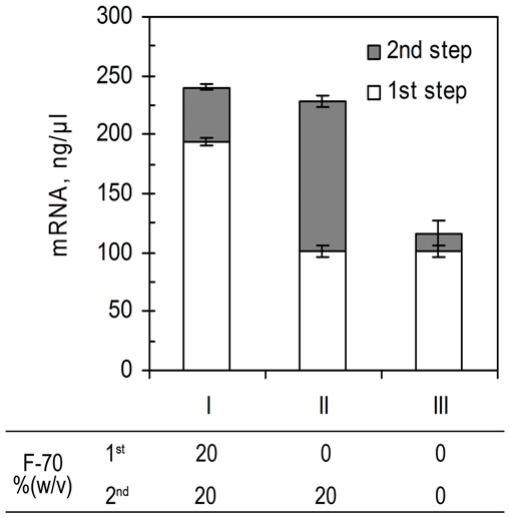
Macromolecular crowding effects on the thermodynamics of *in vitro* transcription. *In vitro* transcriptions were split into two steps, 1^st^ step for 3 hr and 2^nd^ step for 5 hr. The macromolecular crowding conditions (emulated with Ficoll-70) for each step are shown in the panel. Each data point represents the mean of triplicate values; error bars indicate the standard deviation from the mean.

The effect of Ficoll-70 on the thermodynamics of transcription was also tested in a two-step transcription (Figure 3). In the 1^st^ step, transcription was terminated at 3 hr and allowed to proceed for a further 5 hr (step 2). Ficoll-70 was either present in both steps (I) or only in step 1 (II) and its effects compared to a dilute control (III). It was interesting to find that transcription was retriggered in the macromolecular crowding environment and could proceed for additional 3–5 hr. The final yield of the Rluc mRNA generated from these two steps was close to that from the reaction under macromolecular crowding conditions in both steps. In contrast, in the absence of the crowding agent in the 2^nd^ step no significant increase in the mRNA yields was observed (Figure 3). This indicated that the crowding agent Ficoll-70 not only accelerated the *in vitro* transcription, but also thermodynamically improved the reaction, resulting in increased yields of the target mRNA.

### In vitro translation under macromolecular crowding conditions

**Figure 4:**
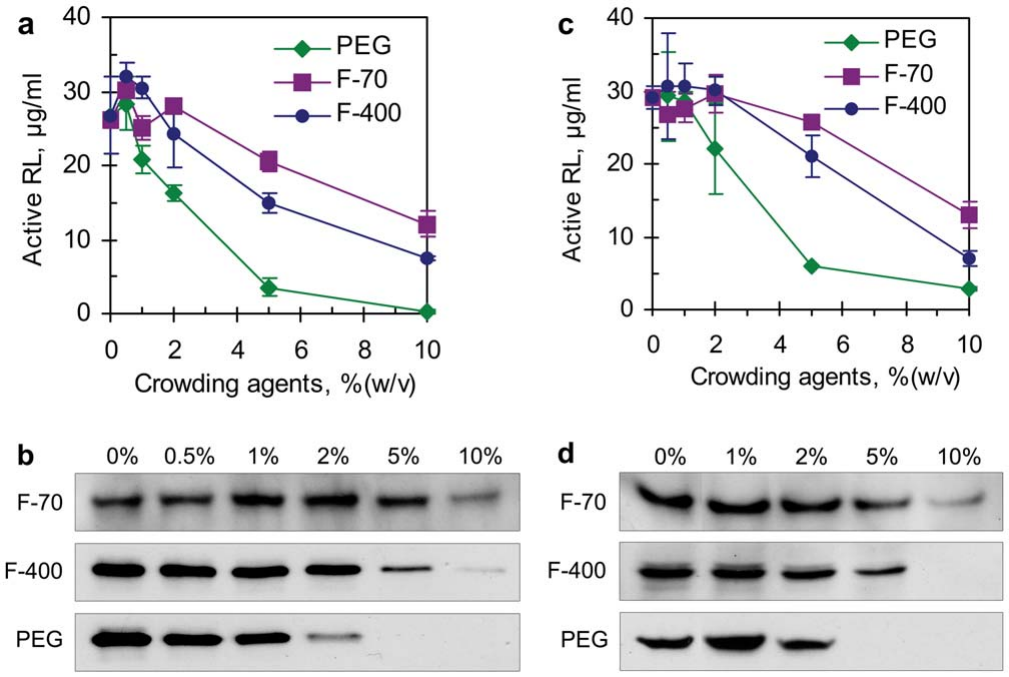
Cell-free protein synthesis under macromolecular crowding conditions. (**a**) Active Rluc protein yields from *in vitro* translation; (**b**) Western blotting detection of the Rluc protein synthesized by *in vitro* translation; (**c**) Active Rluc protein yields from coupled *in vitro* transcription/translation; (**d**) Western blotting detection of the Rluc protein synthesized by coupled *in vitro* transcription/translation. In panel **a** and **c**, each data point is the mean of triplicate values; error bars indicate the standard deviation from the mean.

Translation of Rluc mRNA in the WG-based system was inhibited by all three macromolecular crowding agents at a concentration of >2% (w/v) (Figure 4a). PEG showed the most significant inhibitory effect, decreasing the yield of the active Rluc (measured by luminescence) at a concentration of 1% (w/v). Ficoll, particularly the larger molecule Ficoll-400, slightly enhanced the production of the Rluc at low concentrations (<2%), but became inhibitory at higher concentrations.

The western blots verified that Rluc was produced from the *in vitro* translation (Figure 4b) and enabled us to measure the total Rluc protein yields (both the active and inactive Rluc). As shown in Table 1, the active Rluc accounted for ∼25% of the total protein synthesized by the translation in the absence of the crowding agents, which was consistent with our early findings [42]. However, the percentage of the active Rluc decreased slightly with the increasing concentrations of the crowding agents possibly due to an effect of macromolecular crowding on the correct folding of nascent proteins due to facilitated protein aggregation [35].

**Table 1:** Total Rluc yields and the percentage of active Rluc produced by CFPE.

Crowding agents(%, w/v)	Ficoll-70			Ficoll-400			PEG-8000		
	Total	Active	Active	Total	Active	Active	Total	Active	Active
	(µg/ml)	(µg/ml)	(%)	(µg/ml)	(µg/ml)	(%)	(µg/ml)	(µg/ml)	(%)
*In vitro translation*									
0	106	26.7	25						
1	121	25.1	21	113	30.5	27	96	20.8	22
2	109	27.9	26	106	24.3	23	77	16.3	21
5	92	20.5	22	75	14.8	20	―	3.6	N/A
10	61	12.1	20	38	7.5	20	―	―	N/A
*Coupled in vitro transcription/translation*									
0	115	29.1	25						
1	143	27.6	19	129	30.5	24	153	28.7	19
2	167	29.7	18	111	30.0	27	122	22.1	18
5	136	25.6	19	104	21.0	20	―	5.9	N/A
10	68	13.0	19	―	7.0	N/A	―	2.8	N/A

—: Undetected; N/A: Not Applicable.

### Coupled in vitro transcription/translation under macromolecular crowding conditions

Considering the opposite effects of the crowding agents on transcription (enhancement) and translation (inhibition), we continued to investigate the overall effects of the macromolecular crowding conditions on the protein synthesis in the coupled transcription/translation system. Unexpectedly, the yields of the Rluc protein (both active and total Rluc) consistently decreased in the presence of more than 1.0% (w/v) of PEG or 2.0% (w/v) of two Ficoll molecules, similar to those observed in the *in vitro* translation system (Figure 4c, d). Again, the PEG showed the most significant inhibitory effect; the Rluc protein was hardly detected in the presence of >2.0% of the PEG.

We then examined the Rluc mRNA produced in the coupled transcription/translation solutions by Northern blotting analysis (Figure S1). Surprisingly, the addition of the crowding agents did not increase the mRNA concentrations to the extent expected. The mRNA yields were reduced in the presence of more than 2% (w/v) of Ficoll-70 or Ficoll-400, or more than 5% (w/v) of PEG, which further contributed to the decreased protein yields as shown in Figure 4c. In addition, some truncated mRNA of smaller sizes as well as lager mRNA-protein complexes were also detected in the Northern blotting. The different responses of transcription to the crowding agents between the *in vitro* transcription and the coupled transcription/translation systems should be attributed to the difference in the temperature and salt concentrations, particularly the Mg^2+^ concentrations, of these two systems. The effects of macromolecular crowding on transcription varied significantly with the temperature and salt concentrations of the reaction (Figure S2).

### In vitro translation in PURExpress™ system

Unlike the *in vitro* transcription in a defined sterile solution, the translation was performed in crude cell lysates somewhat like a “black box”, in which many uncharacterized macromolecules and activities, e.g. nonspecific interactions or hydrolytic degradations (by nucleases and proteases), could interfere with the studies of the macromolecular crowding effects. Therefore we turned to a new CFPE system, PURExpress™, that is entirely reconstituted from the defined and purified components necessary for *E. coli* translation [46]. This PURExpress™ system also provided a prokaryotic translation machinery alternative to that of the WG-based system. Ficoll-70 and Ficoll-400 at low concentrations (≤2.5%, w/v) slightly enhanced the protein synthesis in both the *in vitro* translation system and the coupled transcription/translation system, but then inhibited the translations when more crowding agents were added (Figure S3a, b) as was observed for the WG-based system. However, the inhibitory effects in the PURExpress™ system were weaker than in the WG-based system (Figure 4a). In addition, it was interesting to see that in the coupled transcription/translation system, the production of Rluc mRNA was also enhanced by the addition of the crowding agents Ficoll-70 and Ficoll-400 up to the concentration of to 7.5% (w/v), but the highest mRNA yields were detected in the presence of ∼1.0% (w/v) of the Ficoll molecules (Figure S3c), similar to those observed in the WG-based transcription/translation system.

### Two-stage CFPE

**Figure 5:**
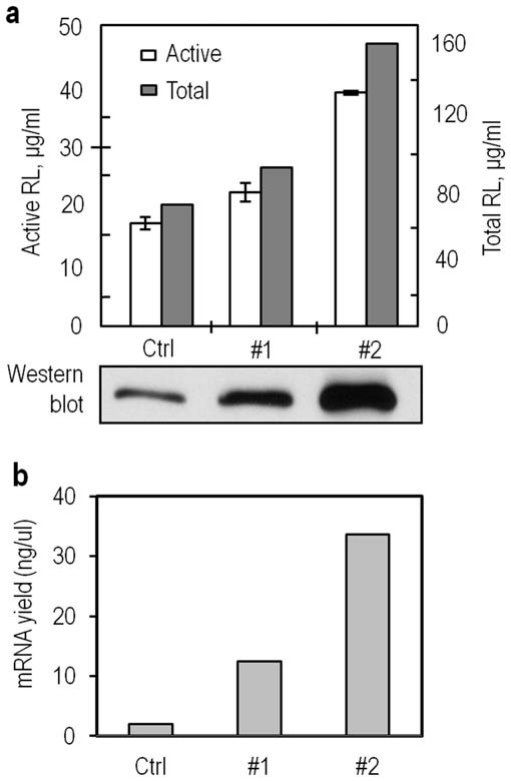
Two-stage CFPE for Rluc protein synthesis from DNA template. (**a**) Protein yields and western blotting detection of the protein synthesized from different CFPE systems. Each data point of the active Rluc is the mean of triplicate values; error bars indicate the standard deviation from the mean; (**b**) Yields of mRNA in different CFPE systems. In both panels, Crtl: coupled CFPE control; #1: control two-stage CFPE without addition of Ficoll-70; #2: Two-stage CFPE with Ficoll-70 added in transcription stage.

Obviously, the *in vitro* transcription and translation processes favored different molecular crowding conditions as well as salt (e.g. Mg^2+^) concentrations. In the coupled CFPE system, the reaction conditions used are usually optimized for the translation such as relatively low macromolecular crowding and high Mg^2+^ concentration, which are suboptimal for transcription process. Currently, establishing a *coupled* CFPE system in which transcription and translation proceeds under different molecular crowding conditions remains technically challenging. However, this can be easily achieved with an *uncoupled* CFPE system. In order to evaluate the potential of CFPE with the macromolecular crowding effects under consideration, we explored a two-stage CFPE (Figure 5), in which transcription and translation were carried out consecutively under their respective optimal conditions: transcription at 37°C in a higher macromolecular crowding solution containing 20% (w/v) of Ficoll-70 while the translation at 30°C in a lower macromolecular crowding solution containing 2% (w/v) of Ficoll-70. Unlike those conventional *uncoupled* CFPE processes where mRNA is first synthesized from a transcription reaction in one tube, purified, and then added to a translation mixture in another tube, this two-stage CFPE features an uncoupled transcription/translation reaction in one tube but at two different volumes: transcription proceeds first in a small volume (5 µl), followed by the addition of translation mixture to a total volume of 50 µl for protein synthesis. Using this technique mRNA synthesis is presumably enhanced by the addition of high concentration of crowding agents. In the subsequent translation step, the high concentrations of crowding agents and salts in transcription stage are subjected to a 1∶10 dilution and thus would not exert inhibitory effects on the translation process. For comparison, a conventional coupled CFPE and a control two-stage CFPE (without addition of crowding agent) were also tested.

With the same amount of DNA template input and the same incubation time (2 hr), the control two-stage CFPE produced slightly (18%) more protein than the coupled CFPE. When a crowding agent, Ficoll-70 was added in transcription stage, then the two-stage CFPE produced 2.2-fold and 1.8-fold more protein than the coupled CFPE and the control two-stage CFPE, respectively (Figure 5a). Obviously, the higher protein yield in the two-stage CFPE resulted from the higher concentration of mRNA available for translation. As seen in Figure 5b, the molecular crowding environment generated 2.7 and 10 times more mRNA than in the absence of Ficoll in the two-step CFPE and the coupled CFPE, respectively We were aware that the mRNA yield detected in the coupled CFPE was actually a dynamic value resulted from constant mRNA generation and degradation. It is noteworthy that the translation process in the coupled CFPE lasted twice as long as those in the two-stage system, but generated lower protein yields because of the low availability of the mRNA template.

### In vitro transcription and translation of Fluc gene under macromolecular crowding environments

**Figure 6:**
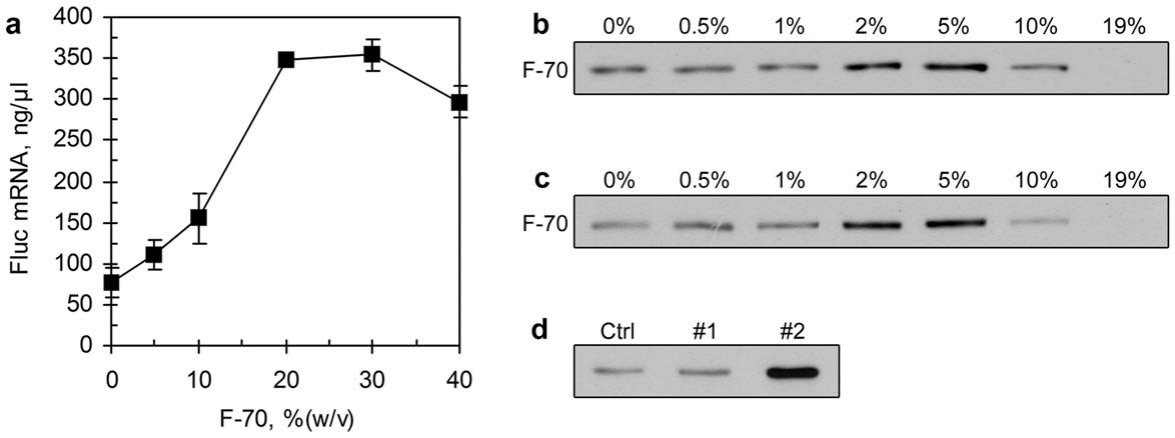
Cell-free synthesis of Fluc protein under macromolecular crowding conditions. (**a**) Effect of crowding agent Ficoll-70 on *in vitro* transcription of the Fluc gene from the pT7CRL-FL template. Transcriptions were incubated for 3 hr; (**b**) Western blotting detection of the Fluc protein synthesized by *in vitro* translation with different concentrations of Ficoll-70. (**c**) Western blotting detection of the Fluc protein synthesized by coupled *in vitro* transcription/translation with different concentrations of Ficoll-70; (**d**) Western blotting detection of the Fluc protein synthesized in two-stage CFPE. Lane Crtl: coupled CFPE control; Lane #1: control two-stage CFPE without addition of Ficoll-70; Lane #2: two-stage CFPE with Ficoll-70 added in transcription stage.

In order to examine if the results obtained from the template of pIVEX1.3-RL also apply to another unrelated template, we further investigated the effects of crowding agent on *in vitro* transcription and translation using the pT7CRL-FL template (Figure 1b). This vector contains a Fluc gene (∼1.6 kb) which is much larger than the Rluc gene (∼0.9 kb). As found for the Rluc gene, transcription of Fluc gene was enhanced by the addition of Ficoll-70 (Figure 6a). The mRNA yield was increased by 5-fold when the Ficoll-70 concentration was increased from 0% to 20–30% (w/v). *In vitro* translation and coupled transcription/translation showed that the expressions of Fluc protein were improved by the addition of 2% to 5% (w/v) of Ficoll-70, but inhibited in the presence of >10% (w/v) (Figure 6b, c), which is also similar to that obtained with the expression of Rluc protein. The two-stage CFPE with 20% (w/v) Ficoll-70 added in transcription stage produced 4.4 fold and 3.4 fold more Fluc protein (as assessed by densitometry) than the coupled CFPE and the control two-stage CFPE (without addition of the crowding agent), respectively (Figure 6d), showing more significant improvement in protein expression than with the pIVEX1.3-RL template.

## Discussion

CFPE allows studies on the complex process of genetic message transfer from DNA to protein in which a number of biomolecules and their conformational rearrangements are involved [13], [47], [48]. The addition of macromolecular crowding agents allows mimicry of the excluded volume effect of biological macromolecules in cells. A straightforward approach is to use highly-concentrated extracts of cells that maintain the cellular contents in a natural state. However, it is technically challenging to make such cell extracts without supplementing buffers and adding protective reagents such as reductant DTT. It is also very difficult to study one process in isolation when using such extracts [16]. Alternatively, the macromolecular crowding environments can be created experimentally by adding inert macromolecule such as PEG and Ficoll [22], [49], [50]. Both PEG and Ficoll display excellent biocompatibility and are attractive polymers to mimic those macromolecules present in living cells [51]. In the present study we compared the effects of PEG and two Ficoll polymers on *in vitro* transcription and translation using coupled and two step CFPE in order to find out which of these gave the best protein yields.

It was not surprising to see that transcription was significantly enhanced by these crowding agents considering that the natural transcriptions occur in macromolecular crowding environments. This observation was also consistent with those of other investigations on the macromolecular crowding effects on biochemical reactions involving the DNA/protein association such as DNA replication [52], [53], ligation [30], PCR [54], restriction digestion [55] and nuclease degradation [56]. All of these studies have thus shown that crowding agents dramatically increase the association between enzymes and DNA and facilitated the biomolecular reactions. For the *in vitro* transcription, the enhanced association of T7 RNA polymerase (T7 RNAP) with DNA template under macromolecular crowding conditions could be seen on an agarose gel following electrophoresis of transcription samples (Figure S4). The formation of a large DNA-RNAP-RNA complex resulted from the binding of T7 RNAP to DNA template and the subsequent transcript was more obvious in crowding solutions than in dilute solutions.

The enhanced association of these biomolecules could be attributed to the excluded volume effects of the crowding agents, which increased the effective concentrations of the enzymes and biomolecular reactants [16], [57], and so altered the rates and equilibrium constants of their reactions [31]. Although this could explain the initial enhancement of transcription by all the three crowding agents, Ficoll and PEG behaved in different ways (Figure 2a). However, macromolecular crowding had more complex effects on the rate and equilibrium of biochemical reactions. Except for increasing the thermodynamic activities of the reactants, the crowding agents also increased the viscosity of the solutions, thus would dramatically reduce the diffusion coefficients of biomolecules by factors up to 10-fold [58]. In addition, macromolecular crowding was reported to be unfavorable to enzyme-substrate (DNA) dissociation [53], [58], which would extend the turn-over time of the enzyme and subsequently decrease the reaction rate. Thus, the results presented in Figure 2a were actually the compromise between these opposite effects of the macromolecular crowding. Worthy of notice was the strong inhibitory effect of PEG on transcription at its concentrations of 10% (w/v) and up. PEG is able to dehydrate the protein surface to readily cause protein precipitation [14], [39], [40]. In the presence of more than 10% (w/v) of PEG in transcription solutions, precipitation of proteins or other biomolecules such as ribosomes and nucleic acids was found, as the reaction solutions became slightly cloudy, which should led to the dramatic suppression of transcription process.

It was interesting to see that the macromolecular crowding not only prolonged transcription time (up to 4.5 hr) (Figure 2c) but also retriggered transcription that had terminated in dilute solution (Figure 3). Since there was no extra enzyme added for retriggering the reaction, the T7 RNAP in the original dilute solution must be still active and there was an adequate supply of rNTPs, however, transcription was inhibited by a certain by-product generated from the translation reaction. This inhibitory byproduct has been reported to be inorganic pyrophosphate (PPi) which could sequester the Mg^2+^ by forming a precipitate with Mg^2+^ at a molar composition of 2∶1 of Mg∶PPi [59]. As a cofactor of RNAP, maintenance of Mg^2+^ above 5 mM in the buffer was regarded as critical for efficient transcription [59]. Therefore, transcription could be retriggered by the fresh Mg^2+^ supplemented together with the transcription buffer. However, in the absence of crowding agent, the retriggered transcription terminated quickly and yielded much less (∼1/10) new Rluc mRNA than the retriggered transcription containing 20% (w/v) of Ficoll-70 (Figure 3). Obviously, the crowding agent played a more important role than the Mg^2+^ in extending the reaction in this case. The conformation of the T7 RNAP might be altered under the macromolecular crowding environment so that the enzyme activity was not as dependent on the Mg^2+^ as that in dilute solution; as such the inhibitory effect of the PPi byproduct could be substantially alleviated.

Compared with transcription and many other enzymatic reactions that have been investigated under macromolecular crowding conditions, translation is a much more sophisticated process in which more than 100 molecules including ribosome, mRNA, tRNAs, tRNA synthetases and amino acids, etc. work in concert to generate polypeptides from mRNA templates [46], [47]. Although the cell-free PURExpress® system has been successfully developed, crude cell extracts, often derived from *Escherichia coli*, rabbit reticulocytes or wheat germ (WG) have still been used in most CFPE applications. We focused on the eukaryotic WG-based system since it contains less inhibitors and allows ready application to high-throughput expression [60]. While the *E. Coli*-based translation systems are often supplemented with 2–5% (w/v) of PEG [14], [61], the WG-based systems are free from crowding agents [11], [62]. We were aware that, even without the addition of any crowding agents, the current translation systems have already been carried out in relatively crowding environments containing ∼5% (w/v) of macromolecules (Table S1). The supplementation of 0.5–10% of synthetic crowding agents (PEG or Ficoll) in this research increased the concentrations of macromolecules up to ∼15% (w/v), which was still less than those of living cytoplasm (30–40%, w/v). Obviously, the observed translation inhibition was not due to the macromolecular crowding effects. Instead, other factors relevant to the added crowding agents are most possibly responsible for the inhibitory effects.

Presence of more than 5% (w/v) of PEG in the *E-Coli* S30-based translation solutions was earlier reported to induce protein precipitation [14], [39], [40], leading to dramatic reduction of the protein synthesis. Similarly, the PEG-induced protein precipitation was also observed in the WG-based translation system, even in the presence of low concentrations of PEG (2–3%, w/v). Obviously, this could cause the rapid reduction of the protein production shown in Figure 4. However, this mechanism could not be used to explain the translation inhibition caused by the Ficoll molecules. Even in the presence of 20% (w/v) of Ficoll-70 and Ficoll-400, there was no protein precipitation observed. Also the molecular size of these two Ficoll molecules did not make big difference in terms of their effects on translation (Figure 4). Some specific chemical properties of these molecules, e.g. non-specific binding to translation components [16], [63], must be responsible for their inhibitory effects. In addition, the diffusion limit of biomolecules in macromolecular crowding solutions may also reduce the translation efficiency [64].

The purpose of setting up the two-stage CFPE (Figure 5) was to explore the potential of this CFPE for improved protein synthesis with the macromolecular crowding effects being integrated. Due to the limit of the volume in transcription that was subject to a 10-fold dilution later for optimal translation, the DNA template input in the two-stage CFPE system was ∼10-fold lower than in the coupled CFPE carried out earlier (Figure 4). However, because of the enhanced transcription by the macromolecular crowding effects, the final protein yield of the two-stage CFPE was comparable to those of the coupled CFPE fed with10-fold more DNA templates, indicating that the efficiency of the CFPE was significantly enhanced in the two-stage system. This has implication in functional genomics and proteomics as it will allow underrepresented genetic information (DNA templates) to be efficiently expressed for functional testing.

The macromolecular crowding effects on CFPE were subsequently confirmed by cell-free synthesis of a larger Fluc protein using an unrelated template pT7CRL-FL. In fact it was found that the crowding agent Ficoll-70 improved transcription and two-stage CFPE more significantly with the pT7CRL-FL template than with the pIVEX1.3-RL template (Figure 6). This indicated that while the macromolecular crowding effects could be generally applicable to CFPE, their extents were related to the size of target gene or its encoding protein.

Finally, it is worth pointing out that only the macromolecular crowding effects on CFPE were discussed in this research. In fact, the efficiency of CFPE is also limited by other factors such as energy supply and inhibitory by-products generated from translation process [65], [66], which are beyond the discussion of this work. In summary, the CFPE was substantially affected by the macromolecular crowding environments emulated by those synthetic crowding agents. While the *in vitro* transcription was significantly enhanced by the high concentrations of crowding agents, particularly Ficoll-70, the translation was generally inhibited. A two-stage CFPE integrating the macromolecular crowding effects could improve the efficiency of CFPE. Our results have profound implications in system and synthetic biology and will allow us to better reproduce the gene transcription and translation process in an *in vitro* setting.

## Materials and Methods

### Molecular crowding agents

PEG-8000 (molecular weight: ∼8 kDa) was purchased from Fisher (Pittsburg, PA). Ficoll-70 and Ficoll-400 (molecular weight: ∼70 kDa and ∼400 kDa, respectively) were purchased from Spectrum Chemicals & Laboratory Products (Gardena, CA). A 50% (w/v) stock solution of each crowding agent was prepared in nuclease-free water before being used in experiments.

### DNA templates

The cell-free protein expression vector pIVEX1.3-RL harboring the Rluc gene (Figure 1a) was constructed from the plasmid pIVEX1.3WG (Roche) as previously described [42], [67]. The expression vector pT7CRL-FL harboring the Fluc gene (Figure 1b) was obtained from Promega (provided as a Fluc T7 Control DNA template). For both vectors, gene expression was under the control of a T7 promoter. The plasmid pIVEX1.3-RL and pT7CRL-FL was linearized with *Apa* I and *EcoICR* I, respectively, and then purified with phenol: chloroform: isoamyl alcohol extraction and ethanol precipitation before being used for *in vitro* transcription.

### *In vitro* transcription and mRNA quantification

Transcription was carried out by mixing 0.4 µl 50× transcription buffer (2 M Tris-HCl pH 8.0, 0.3 M MgCl_2_, 0.1 M spermidine, 0.5 M NaCl), 0.2 µl 1 M DTT, 0.5 µl RNasin® Plus RNase Inhibitor (40 U/µl, Promega, WI), 0.5 µl (for transcription of Rluc gene) or 1.2 µl (for transcription of Fluc gene) ribonucleotide triphosphates (rNTPs, 80 mM each, New England BioLabs), 0.4 µg pIVEX1.3-RL plasmid or 0.2 µg pT7CRL-FL plasmid, 0.5 µl T7 RNA polymerase (RNAP) (50 U/µl, New England BioLabs) and nuclease-free water or crowding agents to a final volume of 20 µl, and incubated at 37°C for various period of time. After the reaction, the DNA template was removed by digestion with 0.5 µl DNase I (2,000 U/ml, New England BioLabs) at 37°C for 15 min. Total mRNA was then quantified with the Quant-iT™ RiboGreen® RNA Reagent and Kit (Invitrogen, CA) according to the manufacturer's instructions.

For two-step transcription, the first step was initiated by mixing 0.2 µl 50× transcription buffer, 0.1 µl 1 M DTT, 0.25 µl RNasin, 0.25 µl rNTPs, 0.4 µg pIVEX1.3-RL DNA template, 0.44 µl RNAP and nuclease-free water and/or Ficoll-70 to a final volume of 10 µl, and incubated at 37°C for 3 hr. In the second step, the reactions were supplemented with 0.2 µl 50× transcription buffer, 0.1 µl 1 M DTT, 0.25 µl RNasin, 0.25 µl rNTPs and nuclease-free water and/or Ficoll-70 to a final volume of 20 µl, and incubated at 37°C for further 5 hr.

### *In vitro* translation and coupled transcription/translation

Translation in the WG-based system was carried out by mixing 10 µl WG extract (Promega), 0.5 µl amino acid mixture (1 M each), 0.4 µl RNasin®, 0.45 µg Rluc mRNA or Fluc mRNA (transcripted from the pIVEX1.3-RL template and the pT7CRL-FL template, respectively), and nuclease-free water or crowding agents to a final volume of 20 µl, and incubated at 30°C for 2 hr. Protein synthesis with the TNT® Coupled Transcription/Translation system (Promega) was carried out by mixing 10 µl WG lysate, 0.8 µl TNT® reaction buffer, 0.5 µl amino acid mixture (1 M each), 0.4 µl RNasin, 0.5 µl T7 RNAP, 2 µg pIVEX1.3-RL template or pT7CRL-FL template, and nuclease-free water or crowding agents to a final volume of 20 µl, and incubated at 30°C for 2 hr.

### Agarose gel electrophoresis and Northern blotting

The samples from the *in vitro* transcription were treated with DNase I; the samples from the coupled transcription/translation were treated with DNase I first, and then extracted with phenol/chloroform solution and ethanol precipitation. Then 5 µl of each sample was mixed with 3 volumes of formaldehyde loading dye (New England BioLabs) and incubated at 65°C for 15 min. The samples were then electrophoresed on a 1% agarose/formaldehydel gel. Post-staining was done with SYBR® Green II RNA Gel Stain (Invitrogen, CA). Images were captured with a Foto/Analyst PC Image (Fotodyne, WI).

For Northern blotting, a digoxigenin (DIG)-labeled DNA probe was synthesized by random primed labeling with a DIG-High Prime Labeling and Detection kit (Roche) using the Rluc gene (*Nco*I-*Xma*I fragment of the plasmid pIVEX1.3-RL) as a template. After agarose gel electrophoresis, RNA was transferred to a positively charged nylon membrane (Ambion, TX). The methods for RNA transfer, pre-hybridization, hybridization with a DIG-labeled probe, low stringency washing and high stringency washing were carried out following the NorthernMax® protocol (Ambion, TX). The DIG label was then detected by chemiluminescence (exposed to X-ray films) according to the manufacturer's manual (Roche).

### SDS-PAGE and Western blotting

Five µl each of the *in vitro* translation solutions was separated on a 12% SDS polyacrylamide gel (SDS-PAGE). The proteins were then electrically transferred to a PVDF membrane for Western blotting analysis. The primary antibody was a rabbit anti-*Renilla* luciferase or a rabbit anti-*Firefly* luciferase polyclonal antibody (MBL International Corp, MA) and the secondary antibody was a goat anti-Rabbit IgG (whole molecule) conjugated with Peroxidase (Jackson ImmunoResearch Laboratories, Inc., PA). The protein bands on PVDF membranes were visualized on X-ray films following exposure of the membranes to SuperSignal® West Pico Chemiluminescent Substrate (Thermo Scientific, IL).

### Protein quantification

Total Rluc protein was quantified based on Western blotting analysis as before [42]. The Rluc standard (Prolume Ltd, AZ) was used to construct the calibration curve. The samples were electrophoresed on the same gel with varying amounts of Rluc standard, and then the concentration of the expressed Rluc was determined by comparison of the band intensities and the calibration curve. Functional Rluc protein was determined using a *Renilla* luciferase Assay System kit (Promega, MI) according to the manufacturer's manual. The relative luminescence unit (RLU) for each sample was measured with a Modulus™ system (Turner Biosystems Inc., CA). The measured RLU was converted to weight concentration according to a calibration curve obtained with the Rluc standard.

## Supporting Information

Figure S1 **Northern blotting analysis of the Rluc mRNA produced in coupled transcription/translation.** Each reaction solution (after 2 hr incubation) was treated with DNase I to remove DNA template. The Rluc mRNA was then extracted with phenol/chloroform and ethanol precipitation before being subjected to electrophoresis.
(DOCX)

Figure S2 **Effects of PEG-8000 (PEG), Ficoll-70 (F-70) and Ficoll-400 (F-400) on** ***in vitro*** **transcription under different conditions.** (**a**) In transcription buffer at 30°C; (**b**) In coupled transcription/translation buffer at 30°C; (**c**) In transcription buffer at 37°C; (**d**) In coupled transcription/translation buffer at 37°C. In all panels, each data point is the mean of triplicate values; error bars indicate the standard deviation from the mean.
(DOCX)

Figure S3 **Cell-free protein expression under macromolecular crowding conditions in the PURExpress™ system.** (**a**) Active Rluc protein yields from *in vitro* translation; (**b**) Active Rluc protein yields from coupled *in vitro* transcription/translation; (**c**) Northern blotting analysis of the Rluc mRNA in coupled *in vitro* transcription/translation (after 2 hr incubation).
(DOCX)

Figure S4 **Agarose gel separation of the** ***in vitro*** **transcription solutions with (+) or without (−) Ficoll-70 (20%, w/v) added.** Two control samples, one without T7 RNAP (control **#1**) and the other without rNTPs (control **#2**) were loaded for comparison. The band corresponding to the DNA/RNA-RNAP complex was absent in control **#1**, and appeared in control **#2** but was not as dense as that observed in the samples (at 10 min).
(DOCX)

Table S1 **Estimation of the macromolecular concentrations of different** ***in vitro*** **translation solutions.**
(DOCX)
