# Supplementary material for: Cell-Free Protein Expression under Macromolecular Crowding Conditions
Source: PLoS One. 2011 Dec 8;6(12):e28707. doi: 10.1371/journal.pone.0028707 (PMC3234285; doi:10.1371/journal.pone.0028707)
Supplement: Figure S1 — Northern blotting analysis of the Rluc mRNA produced in coupled transcription/translation. Each reaction solution (after 2 hr incubation) was treated with DNase I to remove DNA template. The Rluc mRNA was then extracted with phenol/chloroform and ethanol precipitation before being subjected to electrophoresis. (DOCX) [file pone.0028707.s001.docx]

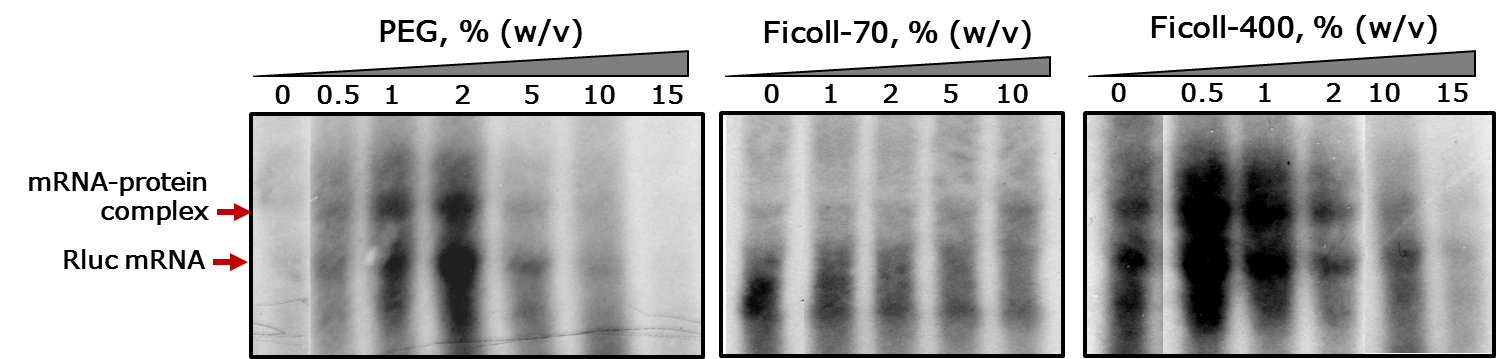


**Figure S1.** **Northern blotting analysis of the Rluc mRNA produced in coupled transcription/translation**. Each reaction solution (after 2 hr incubation) was treated with DNase I to remove DNA template. The Rluc mRNA was then extracted with phenol/chloroform and ethanol precipitation before being subjected to electrophoresis.
