# Supplementary material for: Cell-Free Protein Expression under Macromolecular Crowding Conditions
Source: PLoS One. 2011 Dec 8;6(12):e28707. doi: 10.1371/journal.pone.0028707 (PMC3234285; doi:10.1371/journal.pone.0028707)
Supplement: Figure S2 — Effects of PEG-8000 (PEG), Ficoll-70 (F-70) and Ficoll-400 (F-400) on in vitro transcription under different conditions. (a) In transcription buffer at 30°C; (b) In coupled transcription/translation buffer at 30°C; (c) In transcription buffer at 37°C; (d) In coupled transcription/translation buffer at 37°C. In all panels, each data point is the mean of triplicate values; error bars indicate the standard deviation from the mean. (DOCX) [file pone.0028707.s002.docx]

**a b**

**c d**

**Figure S2**. **Effects of PEG-8000 (PEG), Ficoll-70 (F-70) and Ficoll-400 (F-400) on *in vitro* transcription under different conditions:** (**a**) In transcription buffer at 30 °C; (**b**) In coupled transcription/translation buffer at 30 °C; (**c**) In transcription buffer at 37 °C; (**d**) In coupled transcription/translation buffer at 37 °C. In all panels, each data point is the mean of triplicate values; error bars indicate the standard deviation of the mean

As seen in panel **a** and **c**, even in the simple *in vitro* transcription buffer, the effects of crowding agents Ficoll-70 and Ficoll-400 on transcription were very much dependent on temperature. At lower temperature (30 °C) where *in vitro* translations were often performed, transcriptions were not affected as much by these Ficoll molecules as the reactions were at 37°C. In contrast, the effects of PEG on transcriptions were remarkable but did not vary much with temperature. In the coupled transcription/translation buffer with a high Mg^2+^ concentration, the measured mRNA yields were much lower than those found in the transcription buffer. The addition of Ficoll molecules did not significantly enhance transcription at either 30°C or 37°C (panel **b** and **d**), but the addition of PEG strikingly improved the mRNA yields at relatively low concentrations (<5%, w/v), corroborating those found in Northern blotting analysis (Supplemental Figure 1).
