# Supplementary material for: Cell-Free Protein Expression under Macromolecular Crowding Conditions
Source: PLoS One. 2011 Dec 8;6(12):e28707. doi: 10.1371/journal.pone.0028707 (PMC3234285; doi:10.1371/journal.pone.0028707)
Supplement: Figure S3 — Cell-free protein expression under macromolecular crowding conditions in the PURExpress™ system. (a) Active Rluc protein yields from in vitro translation; (b) Active Rluc protein yields from coupled in vitro transcription/translation; (c) Northern blotting analysis of the Rluc mRNA in coupled in vitro transcription/translation (after 2 hr incubation). (DOCX) [file pone.0028707.s003.docx]

**a**  **b**


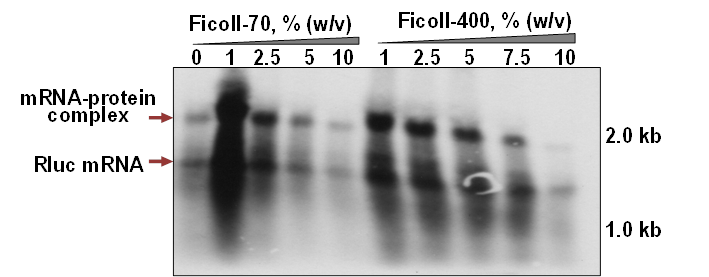
 **c**

**Figure S3. Cell-free protein expression under macromolecular crowding conditions in the PURExpress™ system**. (**a**) Active Rluc protein yields from *in vitro* translation; (**b**) Active Rluc protein yields from coupled *in vitro* transcription/translation; (**c**) Northern blotting analysis of the Rluc mRNA in coupled *in vitro* transcription/ translation (after 2 hr incubation).

**Methods:**

The PURExpress™ *In vitro* Protein Synthesis Kit was purchased from New England Biolabs Inc. (Ipswich, MA). *In vitro* translation was carried out by mixing 4 µl solution A, 3 µl solution B, 0.5 µl RNasin, 700 ng Rluc mRNA (transcripted from pIVEX2.3-RL template, Roche) and nuclease-free water or crowding agents to a final volume of 20 µl, and incubated at 37 °C for 2 hr. *In vitro* coupled transcription/translation was carried out by mixing 4 µl solution A, 3µl solution B, 0.5 µl RNasin, 500 ng pIVEX2.3-RL template and nuclease-free water or crowding agents to a final volume of 10 µl, and incubated at 37 °C for 2 hr. For Northern blotting analysis, each reaction solution was treated with DNase I to remove DNA template. The Rluc mRNA was further extracted with phenol/chloroform and ethanol precipitation before being subjected to electrophoresis.
