# Supplementary material for: Cell-Free Protein Expression under Macromolecular Crowding Conditions
Source: PLoS One. 2011 Dec 8;6(12):e28707. doi: 10.1371/journal.pone.0028707 (PMC3234285; doi:10.1371/journal.pone.0028707)
Supplement: Figure S4 — Agarose gel separation of the in vitro transcription solutions with (+) or without (−) Ficoll-70 (20%, w/v) added. Two control samples, one without T7 RNAP (control #1) and the other without rNTPs (control #2) were loaded for comparison. The band corresponding to the DNA/RNA-RNAP complex was absent in control #1, and appeared in control #2 but was not as dense as that observed in the samples (at 10 min). (DOCX) [file pone.0028707.s004.docx]

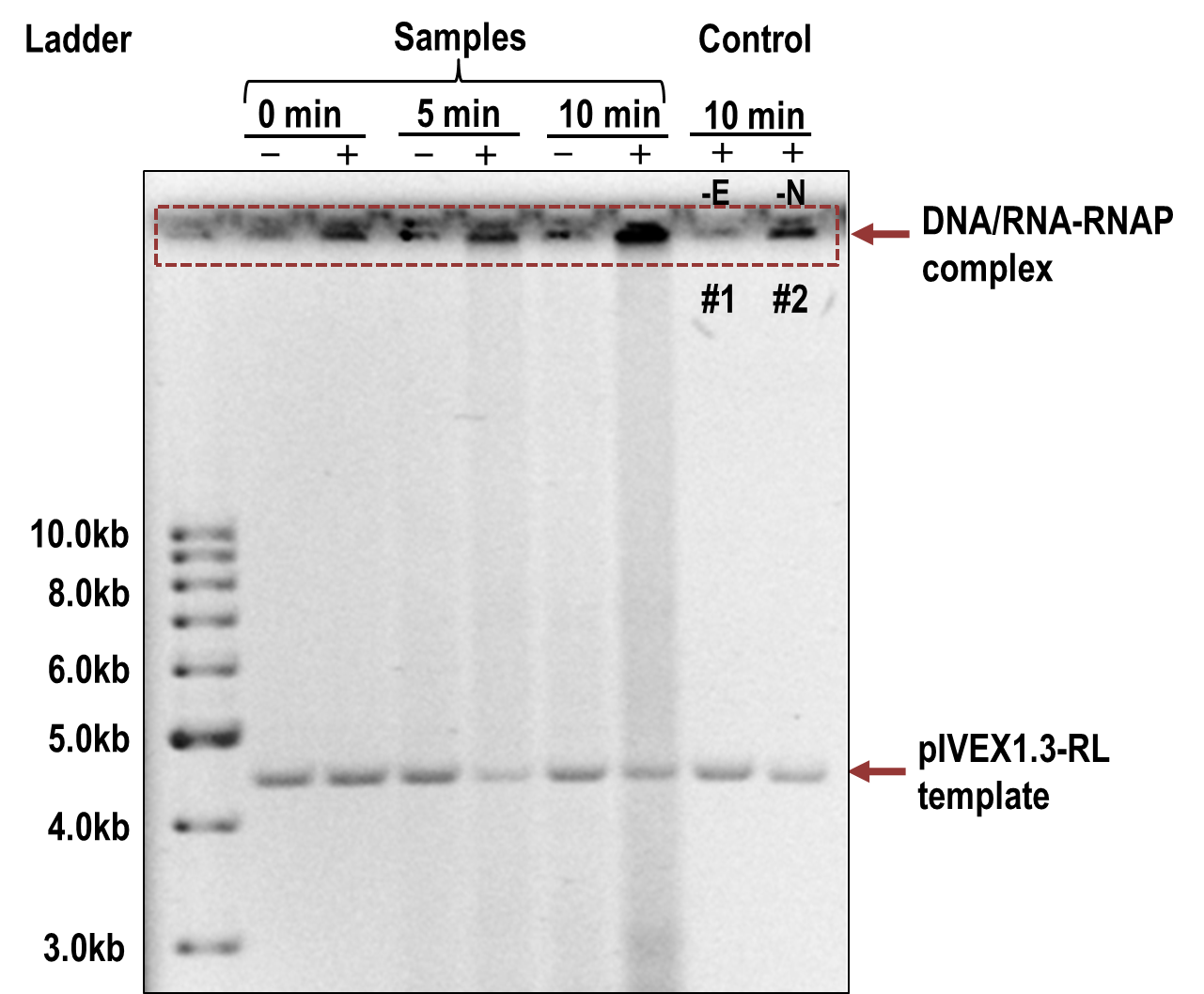


**“+”: Fiocll-70 added; “-“: no Ficoll-70 added**

**“-E”: no enzyme (T7 RNAP) added; “-N”: no rNTPs added**

**Figure S4**. **Agarose gel separation of the *in vitro* transcription solutions with (+) or without (-) Ficoll-70 (20%, w/v) added**. Two control samples, one without T7 RNAP (control **#1**) and the other without rNTPs (control **#2**) were loaded for comparison. The band corresponding to the DNA/RNA-RNAP complex was absent in control **#1**, and appeared in control **#2** but was not as dense as that observed in the samples (at 10 min).

**Method:**

*In vitro* transcription was carried out by mixing 0.2 µl of 50X transcription buffer, 0.1 µl of 1M DTT, 0.25 µl of RNasin, 0.25 µl of rNTPs (80 mM each) or 0.25 µl of nuclease-free water for the “-N” sample, 550 ng of pIVEX1.3-RL DNA template, 0.11µl of T7 RNAP or 0.11µl of nuclease-free water for the “-E” sample, and 4 µl of 50% (w/v) Ficoll-70 for reactions “+” or 4 µl of nuclease-free water for reactions “-” to a final volume of 10 µl. Reactions were incubated at 37°C for various periods of time. After reaction, each sample was mixed with 0.5 μl of 20% formaldehyde solution, incubated at room temperature for 10 min, then mixed with 1.12 μl of 1.25 M glycine and incubated at room temperature for 5 min. Before gel electrophoresis, 4 μl of Ficoll-70 was added into each reaction “-“, meanwhile 4 μl of water was added into each reaction “+”, so that all samples had an equal concentration of Ficoll-70. Then, samples were loaded on an agarose gel (0.6% in 1X TEB) and run at 20V, 4℃ for 20 hr in 1X TEB buffer. Post-staining was done with 0.5 μg/ml ethidium bromide. Images were captured with Foto/Analyst PC Image (Fotodyne, WI).
