# Supplementary material for: Cell-Free Protein Expression under Macromolecular Crowding Conditions
Source: PLoS One. 2011 Dec 8;6(12):e28707. doi: 10.1371/journal.pone.0028707 (PMC3234285; doi:10.1371/journal.pone.0028707)
Supplement: Table S1 — Estimation of the macromolecular concentrations of different in vitro translation solutions. (DOCX) [file pone.0028707.s005.docx]

**Table S1. Estimation of the macromolecular concentrations of different *in vitro* translation solutions**

| Macromolecules  % (w/v) | WG-based translation solution^*^ | WG-based Coupled transcription/translation solution^*^ | PURExpress^TM^ system [1] |
| --- | --- | --- | --- |
| Proteins | 3-5 | 3-5 | 0.05 |
| Nucleic acids | 0.2 | 0.2 | 0.34 |
| Carbohydrates | 0.02 | 0.02 | 0 |
| Crowding agents (PEG) | 0 | 0 | 2-3 |
| Total | ~5 | ~5 | ~3 |

*: data obtained from the manuals of the Wheat Germ Extract (Promega, Cat# L4380) and the TnT^®^ T7 Coupled Wheat Germ Extract System (Promega, Cat# L4140)

**Reference:**

1. Shimizu Y, Inoue A, Tomari Y, Suzuki T, Yokogawa T, et al. (2001) Cell-free translation reconstituted with purified components. Nat Biotechnol 19: 751-755.
